# Factors associated to functioning and health in relation to home rehabilitation in Sweden: a non-randomized pre-post intervention study

**DOI:** 10.1186/s12877-021-02360-1

**Published:** 2021-07-06

**Authors:** Anette Johansson, Marie Ernsth Bravell, Eleonor I. Fransson, Sofi Fristedt

**Affiliations:** 1 Health Care Administration, Jönköping Municipality, Jönköping, Sweden; 2 School of Health and Welfare, Jönköping University, Jönköping, Sweden

**Keywords:** Activities of daily living, Mobility, Older adults, Adults, Outcomes

## Abstract

**Background:**

Home rehabilitation is a growing rehabilitation service in many countries, but scientific knowledge of its components and outcomes is still limited. The aim of this study was to investigate; 1) which changes in functioning and self-rated health could be identified in relation to a home rehabilitation program in a population of community-dwelling citizens, and 2) how socio-demographic factors, health conditions and home rehabilitation interventions were associated to change in functioning and self-rated health after the home rehabilitation program.

**Method:**

The sample consisted of participants in a municipal home rehabilitation project in Sweden and consisted of 165 community-dwelling citizens. General Linear Models (ANOVA repeated measures) was used for identifying changes in rehabilitation outcomes. Logistic regressions analysis was used to investigate associations between rehabilitation outcomes and potential factors associated to outcome.

**Result:**

Overall improvements in functioning and self-rated health were found after the home rehabilitation program. Higher frequencies of training sessions with occupational therapists, length of home rehabilitation, and orthopaedic conditions of upper extremities and spine as the main health condition, were associated with rehabilitation outcomes.

**Conclusion:**

The result indicates that the duration of home rehabilitation interventions and intensity of occupational therapy, as well as the main medical condition may have an impact on the outcomes of home rehabilitation and needs to be considered when planning such programs. However, more research is needed to guide practice and policymaking.

## Introduction

The increased proportion of older adults in the population is expected to have an impact on healthcare systems worldwide [1, 2]. This has resulted in the implementation of home rehabilitation programs to promote independence [3–7] and to support citizens to stay-in-place [8–10] in Sweden and many other countries [11]. Home rehabilitation is not a unitary concept and programs can vary in how they are called, and its organization, content and target group [12]. In Sweden, occupational therapists and physiotherapists are the core practitioners in municipal home rehabilitation, and they work in close collaboration with home care services for adults of all ages [12–14], especially for older adults [14]. Different home rehabilitation programs share however common characteristics such as having a limited duration [8, 15], and being intensive, multidisciplinary, person-centred and goal-oriented [8, 15, 16]. Their common aim is to promote independence and support older adults to remain in their homes for as long as possible [8, 9]. Home rehabilitation programs have shown positive effects such as improved ADL skills, self-reported activity performance, quality of life [9, 17, 18] and decreased dependence on home care services [4]. Some studies do however report a lack of long-term effects [19]. Knowledge is also limited as to which components of home rehabilitation programs lead to positive results [12] and if some client groups benefit from the intervention more than others [8]. There are indications that women are more motivated in home rehabilitation after fractures and thus achieve better outcomes than men [20]. The optimal intensity and duration of home rehabilitation programs are also debated [16].

To guide home rehabilitation programs, research is needed on how different factors such as gender, types and length of intervention, and health conditions affect the outcome [15]. Therefore, the aim of this study was to investigate; 1) which changes in functioning and self-rated health could be identified in relation to a home rehabilitation program in a population of community-dwelling citizens, and 2) how socio-demographic factors, health conditions and home rehabilitation interventions are associated to changes in functioning and self-rated health after a home rehabilitation program.

## Method

### The home rehabilitation intervention

**Fig. 1:**
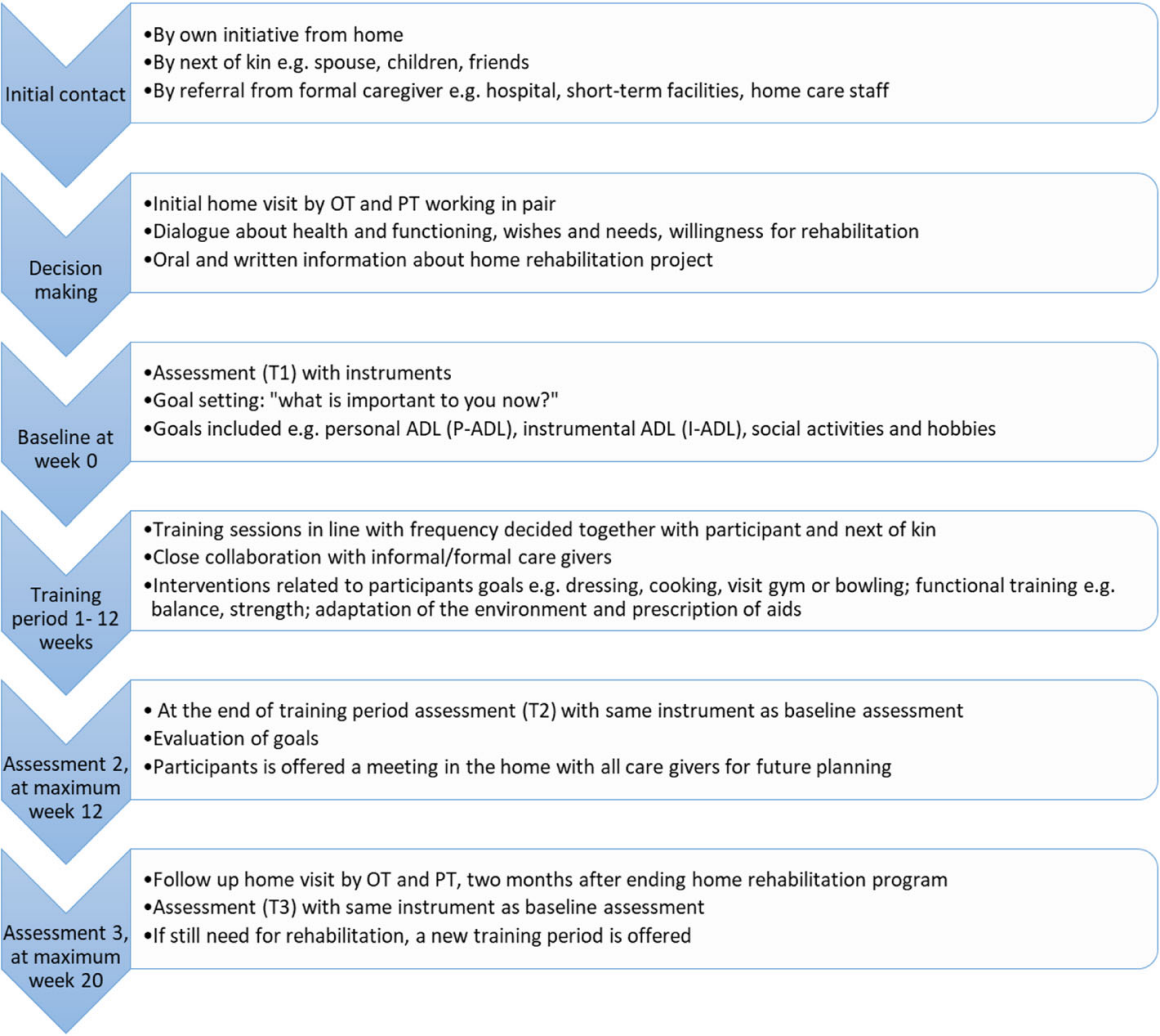
An overview of the home rehabilitation intervention

The present study analyses already collected data derived from a home rehabilitation improvement project in a Swedish municipality with approximately 140,000 citizens. The project was initiated by the municipality to develop their rehabilitation program and was conducted without a control group. The project involved team-based rehabilitation executed by occupational therapists, physiotherapists and rehabilitation assistants in the person’s home and neighbourhood. An overview of the home rehabilitation interventions is presented in Fig. 1. The interventions had a limited time duration and were based on the participants’ own goal. It also included collaboration with other caregivers. For example, home care staff sometimes also assisted with training.

### Participants

Community-dwelling adults that were assessed to need rehabilitation and lived in a selected geographical area (mainly urban and suburban areas of approximately 47,000 inhabitants) were eligible for the municipal home rehabilitation program. Between 2015 and 2019, a total 212 adults participated in the program from different ages and with a range of health conditions. The participants gave informed consent to participate in the municipality project and each received up to 12 weeks of home rehabilitation.

For the purpose of this present study, access to de-identified data was granted by the municipality in line with Swedish laws and regulations [21]. The Swedish Ethical Review Authority (Dnr: 2019–00706) approved that the data would be used for research.

### Data collection

**Fig. 2:**
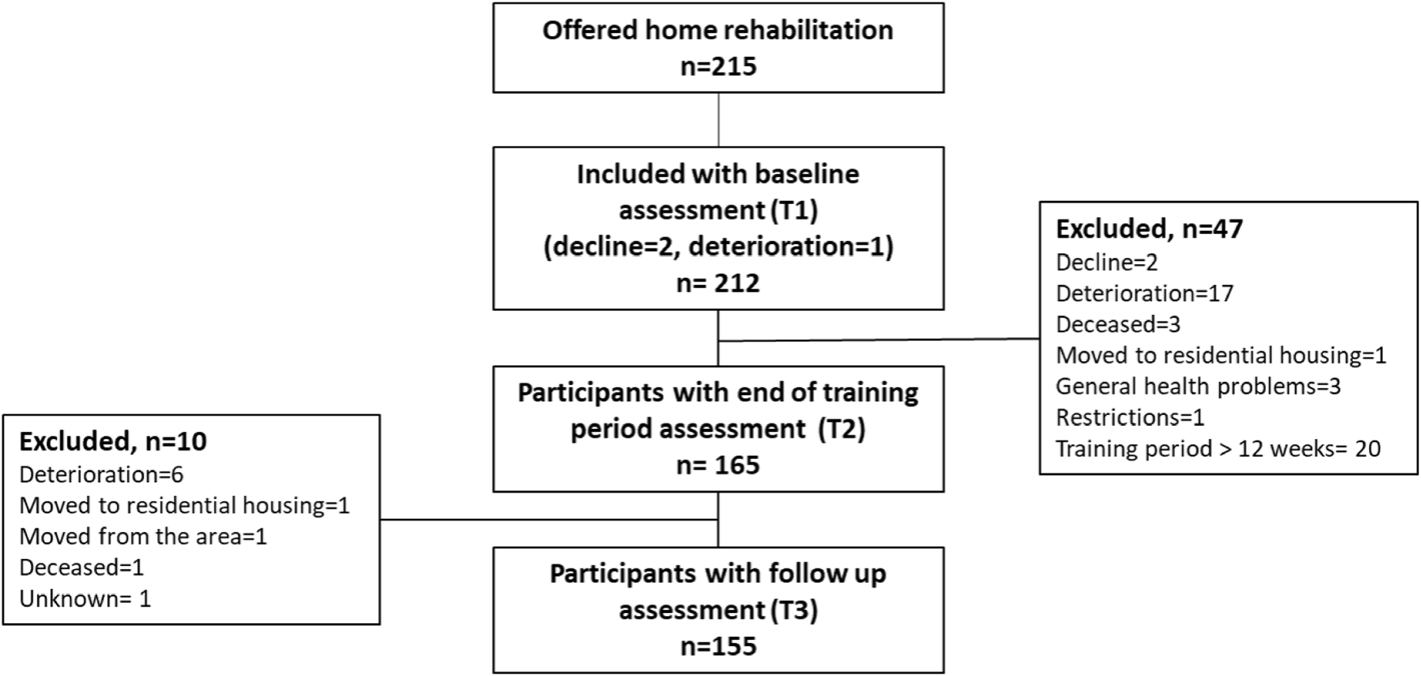
Flowchart of sampling process

During the rehabilitation program, data were collected by the occupational therapists and physiotherapists involved. For this study, the municipality provided the collected and de-identified data from three points of time; baseline (T1), at the end of home rehabilitation intervention (T2) and at 2-month follow-up (T3). A total of 165 participants with data from T1 and T2 were available for analysis for this study (See Fig. 2). Twenty persons did not have a measurement point at T2 due to an extension in their training period and were therefore excluded from our study.

#### Dependent variables: rehabilitation outcomes

##### Functioning in activities of daily living

Sunnaas Activity of Daily Living Index (Sunnaas ADL index) examines the person’s functioning in daily activities [22] and was one of two measures of functioning used. The Sunnaas ADL Index contains ratings of 12 specific daily activities, including eight personal activities of daily living (PADL) and four instrumental activities of daily living (IADL). The level of independence is scored from: 3 = completely independent, 2 = independent but requires aids or adapted environment, 1 = partly dependent of another person and 0 = completely dependent on another person [23]. The Sunnaas ADL index is considered a reliable and valid assessment of function at the activity level [23–25].

For this study, an ADL index was created including the items *feeding, indoor mobility, toilet management, transfer, dressing/undressing, grooming, cooking, bath/shower, housework,* and *outdoor mobility*. The variables *continence* and *communication* were considered less relevant as outcomes of home rehabilitation interventions and were not included in our ADL-index. The score for each included item was dichotomized as 0 = independent (score 3–2) vs. 1 = dependent (score 1–0). The dichotomized scores were summarized, thus giving an ADL index value from 0 to 10. In addition, the ADL index differences between T1 and T3 was calculated and dichotomized to create a variable where 0 = no change/deterioration and 1 = improvement over time. Cronbach’s alpha analysis demonstrated good inter-item reliability of the ADL-index used in this study (0.85).

##### General motor functioning dependence

The General Motor Function assessment scale (GMF) includes 11 mobility functions and 10 upper limb functions [26] and three different subscales: function-related dependence, pain and insecurity. For this study, we used the dependence subscale alone, hereafter GMF dependence. In this measure functioning, degrees of dependence are assessed on a two-point scale for some functions (specified in Table 2); 0 = independent, 1 = help from 1 person/unable, while other functions were measured on a three-point scale; 0 = independent, 1 = help from 1 person, 2 = help from 2 persons/unable (Table 2). The GMF has shown good validity [27, 28], sensitivity [27] and reliability [26].

For this study, two GMF dependence indexes were created. The first index, GMF dependence mobility, included the 11 mobility functions ranging from *turning over in bed* to *climbing stairs* and *transfer outdoors*. Each item was rated 0 = independent (score 0) or 1 = dependent (score 1–2) (Cronbach’s alpha = 0, 70). The second index, GMF dependence upper limb, included the 10 variables for arm movements and grip functions. Items were rated as 0 = independent (score 0) or 1 = dependent (score 1–2) (Cronbach’s alpha = 0, 70). Even here, the difference between T1-T3 was calculated and dichotomized to create a variable in terms of 0 = no change/deterioration vs. 1 = improvement over time.

##### Self-rated health

Self-rated health was measured through *EQ VAS*, which is included in the European Quality of Life Five Dimension Five Level Scale (EQ-5D-5L) [29]. The occupational therapist or physiotherapist performing the rehabilitation asked participants to complete the EQ-5D-5L form, which could then be posted in a pre-stamped envelope addressed to the municipality development unit. In *EQ VAS* in particular, participants assess their health status on the present day using a vertical visual analogue scale ranging from 0 “the worst imaginable health” to 100 “the best imaginable health” [29].

In this study we use the *EQ VAS* as a dependent continuous variable for self-rated health. The EQ-5D-5L has demonstrated satisfactory reliability, validity and responsiveness in older adults [30].

#### Independent variables: factors potentially associated to rehabilitation outcomes

Data on participant characteristics were their age (years), gender (male/female), living situation (living alone/cohabiting), main reasons for home rehabilitation, number of training sessions by rehabilitation staff and length of home rehabilitation in weeks. The main reason for home rehabilitation was defined by the health condition described in professional assessments, diagnoses and/or the person’s own experienced problems leading to the need for rehabilitation.

For this study, data were categorized using information on current diagnosis, medical condition, and affected body part (e.g., mobility difficulties due to hip fracture). If specific information about current diagnosis or medical condition was missing, the person’s medical history was used as a guide to indicate an appropriate category. Seven categories were identified; *conditions in the circulatory and respiratory systems* (e.g., chronic obstructive pulmonary disease, myocardial infarction)*, mobility limitations including fall risk* (condition not specified or e.g., fall trauma, impaired balance)*, multimorbidity and/or frailty* (a complex situation or conditions involving problems in several body regions, e.g., cancer, fibromyalgia), *neurological conditions excl. stroke* (e.g., multiple sclerosis, Parkinson’s disease), *orthopaedic conditions upper extremities and spine* (e.g., vertebral compressions, shoulder fracture), *orthopaedic conditions lower extremities and pelvis* (e.g., femoral amputation, knee osteoarthritis) and *stroke* (acute and post).

### Analyses

General Linear Models (ANOVA repeated measures) were first performed to analyse changes over time in functioning (ADL, GMF dependence) and in self-rated health (EQ VAS) between baseline and at the end of home rehabilitation intervention (T1-T2) and between baseline and 2-month follow-up (T1-T3). Logistic regression analyses were performed to investigate associations between independent variables and the outcomes of functioning and self-rated health in terms of no change/deterioration vs. improvement. Firstly, bivariate analyses were performed to investigate associations for each variable in relation to the outcomes. In the next step, multivariate analyses were performed for all independent variables in the model. Within the variable main reason for rehabilitation, *multimorbidity and/or frailty* were chosen as the reference category.

The number of training sessions that each participant received from an occupational therapist and physiotherapist per week during home rehabilitation were analysed through logistic regression models. Visits by rehabilitation assistants were few and consequently these were not included in the regression models. Potential interaction between intensity and duration was tested by adding an interaction term (number of training session per number of weeks) in each logistic regression model. *P*-values < 0.05 were considered statistically significant. Statistical analyses were performed with IBM SPSS Statistic version 26.

## Results

### Descriptive results

A description of baseline characteristics, home rehabilitation interventions and baseline rehabilitation outcomes are presented in Table 1. The participants had a mean age of 80 years and the majority were women. Half of the participants lived alone, although there is a gender difference as majority of the men lived together with someone, and majority of the women lived alone. *Orthopaedic conditions (lower extremities and pelvis)* were the most frequent reasons for needing home rehabilitation. The participant received an average of 2.8 training sessions per week and the average duration of the training was 8.5 weeks. The rehabilitation outcome scores demonstrated that the participants had a moderate to mild disability at baseline.

**Table 1:** Description of baseline characteristics, home rehabilitation interventions and baseline rehabilitation outcomes

Characteristics	Men (***n*** = 60)	Women (***n*** = 105)	Total (***n*** = 165)
Age in years; mean (SD), min-max	79.2 (9.6), 49–99	81.2 (7.9), 56–96	80.5 (8.6), 49–99
Living situation; n (%)			
Living alone	20 (33.3)	63 (60.0)	83 (50.3)
Cohabiting	40 (66.7)	42 (40.0)	82 (49.7)
Main reasons for home rehabilitation; n (%)			
Conditions in the circulatory and respiratory systems	8 (13.3)	12 (11.4)	20 (12.1)
Mobility limitations incl. fall risk (condition not specified)	5 (8.3)	10 (9.5)	15 (9.1)
Multimorbidity and/or frailty	18 (30.0)	18 (17.1)	36 (21.8)
Neurological conditions (excl. stroke)	6 (10.0)	0 (0.0)	6 (3.6))
Orthopaedic conditions (upper extremities and spine)	3 (5.0)	15 (14.3)	18 (10.9)
Orthopaedic conditions (lower extremities and pelvis)	9 (15.0)	35 (33.3)	44 (26.7)
Stroke (acute and post)	11 (18.3)	15 (14.3)	26 (15.8)
Home rehabilitation intervention; mean (SD), min-max			
Training sessions per week by OT	1.3 (0.6), 0.3–3.6	1.1 (0.5), 0.3–2.3	1.2 (0.6), 0.3–3.6
Training sessions per week by PT	1.8 (0.9), 0.1–4.7	1.3 (0.5), 0–2.5	1.5 (0.7), 0–4.7
Training sessions per week by RA	0.09 (0.3), 0–1.6	0.2 (0.4), 0–1.6	0.1 (0.4), 0–1.6
Total training sessions per week by OT, PT, RA	3.1 (1.3), 1.2–7.5	2.6 (0.9), 1–5.7	2.8 (1.1), 1–7.5
Length of home rehabilitation in weeks	8.4 (2.7), 3–13	8.6 (2.8), 3–13	8.5 (2.7), 3–13
Rehabilitation outcomes; mean (SD), min-max			
ADL index^1^	3.6 (2.2), 0–9	3.4 (2.2), 0–10	3.5 (2.2), 0–10
GMF dependence mobility ^2^	1.6 (1.5), 0–6	1.8 (1.6), 0–8	1.7 (1.6), 0–8
GMF dependence upper limb ^1^	0.6 (1.3), 0–5	0.6 (1.3), 0–5	0.6 (1.3), 0–5
EQ VAS^3^	56.1^4^ (20.5), 10–99	53.1^5^ (17.3), 0–90	54.2^6^ (18.4), 0–99

**Abbreviations:** SD, standard deviation; OT, occupational therapist; PT, physiotherapist; RA, rehabilitation assistant

**Notes:** ^1^index total score, minimum = 0, maximum = 10, lower score is higher degree of independence; ^2^index total score, minimum = 0, maximum = 11, lower score is higher degree of independence; ^3^ total score, 0 = worst health, 100 = best health, higher score is the better outcome, ^4^*n* = 42, ^5^ *n* = 81, ^6^ *n* = 123

### Changes in functioning and self-rated health

The result from the GLM repeated measures is presented in Table 2. There are overall statistically significant improvements in activities of daily living at the end of the home rehabilitation intervention (T2) and at the two-month follow-up (T3). The change in ability to eat is only statistically significant between T1 and T3. The ADL index demonstrated significant improvements between both T1 and T2 (3.47; 2.20, a difference of 1.27) and between T1 and T3 (3.47; 2.10, a difference of 1.37). The GMF dependence mobility index showed statistically significant improvements between both T1 and T2 (1.69; 0.85, a difference of 0.84) and between T1 and T3 (1.69; 0.66, a difference of 1.03). The GMF dependent upper limb index demonstrated statistically significant improvement between both T1 and T2 (0.58; 0.43, a difference of 0.15) and between T1 and T3 (0.58; 0.40, a difference of 0.18). *EQ VAS* showed significant improvement between both T1 and T2 (55.11; 62.62, a difference of 7.51) and between T1 and T3 (55.11; 66.42, a difference of 11.31).

**Table 2:** Changes in functioning and self-rated health over time in relation to home rehabilitation

	Baseline T_**1**_	End of home rehabilitation T_**2**_	General Linear Model T_**1**_ – T_**2**_	2-month follow-up T_**3**_	General Linear Model T_**1**_ – T_**3**_
	Mean (SD)	Mean (SD)	*P-*value	Mean (SD)	*P-*value
**Functioning**					
**Activity of Daily Living**^1^	*n* = 165	*n* = 165		*n* = 155	
Eating (0–3)	2.90 (0.34)	2.92 (0.32)	.083	**2.92 (0.32)**	**.045**
Indoor mobility (0–3)	**2.11 (0.60)**	**2.31 (0.57)**	**< .001**	**2.38 (0.58)**	**< .001**
Toilet management (0–3)	**2.11 (0.65)**	**2.24 (0.58)**	**< .001**	**2.25 (0.59)**	**< .001**
Transfer (0–3)	**2.28 (0.71)**	**2.51 (0.62)**	**< .001**	**2.55 (0.62)**	**< .001**
Dressing/undressing (0–3)	**2.19 (0.89)**	**2.50 (0.75)**	**< .001**	**2.48 (0.79)**	**< .001**
Grooming (0–3)	**2.55 (0.65)**	**2.70 (0.59)**	**< .001**	**2.71 (0.59)**	**< .001**
Cooking (0–3)	**1.36 (1.12)**	**1.85 (1.07)**	**< .001**	**1.91 (1.10)**	**< .001**
Bath/shower (0–3)	**1.42 (0.81)**	**1.75 (0.85)**	**< .001**	**1.80 (0.87)**	**< .001**
Housework (0–3)	**0.82 (0.76)**	**1.15 (0.87)**	**< .001**	**1.27 (0.96)**	**< .001**
Outdoor mobility (0–3)	**1.05 (0.70)**	**1.63 (0.64)**	**< .001**	**1.67 (0.64)**	**< .001**
ADL index^2^	**3.47 (2.19)**	**2.20 (2.11)**	**< .001**	**2.10 (2.20)**	**< .001**
**GMF dependence mobility** ^2^	*n* = 158	*n* = 158		*n* = 149	
Turn around when lying in bed (0–2)	**0.09 (0.33)**	**0.04 (0.19)**	**.020**	**0.02 (0.14)**	**.012**
Sit up from recumbent position (0–2)	**0.04 (0.24)**	**0.02 (0.18)**	**.045**	**0.01 (0.16)**	**.025**
Lie down from a sitting position (0–2)	0.03 (0.21)	0.01 (0.16)	.083	0.01 (0.16)	.083
Transfer from bed to chair (0–2)	0.05 (0.27)	0.03 (0.24)	.083	0.03 (0.24)	.083
Touch left big toe (0–1)	**0.23 (0.58)**	**0.16 (0.50)**	**.007**	0.18 (0.60)	.103
Touch right big toe (0–1)	**0.27 (0.62)**	**0.20 (0.60)**	**.007**	0.20 (0.63)	.059
Stand up from a sitting position (0–2)	**0.08 (0.31)**	**0.05 (0.25)**	**.045**	**0.05 (0.24)**	**.045**
Stand more than 10 s (0–2)	0.04 (0.24)	0.03 (0.19)	.083	**0.01 (0.16)**	**.025**
Transfer indoors 10 m (0–2)	**0.08 (0.29)**	**0.02 (0.14)**	**.006**	**0.01 (0.12)**	**.007**
Climb stairs up/down 7 steps (0–2)	**0.66 (0.74)**	**0.26 (0.56)**	**< .001**	**0.24 (0.56)**	**< .001**
Transfer outdoors 25 m (0–2)	**0.60 (0.62)**	**0.29 (0.47)**	**< .001**	**0.22 (0.43)**	**< .001**
GMF dependence mobility index^2^	**1.69 (1.54)**	**0.85 (1.30)**	**< .001**	**0.66 (1.11)**	**< .001**
**GMF dependence upper limb** ^2^					
Move left hand to mouth (0–1)	0.04 (0.19)	0.03 (0.16)	.158	0.03 (0.16)	.158
Move right hand to mouth (0–1)	0.02 (0.14)	0.02 (0.14)	1.00	0.02 (0.14)	1.00
Move left hand to head (0–1)	0.06 (0.23)	0.04 (0.19)	.083	0.04 (0.20)	.083
Move right hand to head (0–1)	0.09 (0.35)	0.06 (0.24)	.096	0.06 (0.24)	.059
Move left hand on back (0–1)	0.09 (0.35)	0.05 (0.22)	.052	**0.04 (0.20)**	**.032**
Move right hand on back (0–1)	0.09 (0.35)	0.07 (0.26)	.181	0.06 (0.24)	.319
Greeting grip with left hand (0–2)	**0.11 (0.37)**	**0.06 (0.26)**	**.032**	**0.05 (0.24)**	**.007**
Greeting grip with right hand (0–2)	0.08 (0.32)	0.06 (0.26)	.207	0.05 (0.25)	.132
Pinch grip with left hand (0–2)	0.06 (0.31)	0.04 (0.26)	.181	0.05 (0.27)	.181
Pinch grip with right hand (0–2)	0.04 (0.21)	0.03 (0.18)	.158	0.03 (0.22)	.319
GMF dependent upper limb index^2^	**0.58 (1.29)**	**0.43 (1.16)**	**.008**	**0.40 (1.14)**	**.001**
**Self-rated health**	*n* = 106	*n* = 106		*n* = 91	
EQ VAS^1^	**55.11 (17.82)**	**62.62 (17.85)**	**< .001**	**66.42 (17.01)**	**< .001**

**Notes:** ^1^improvement shows in higher score, ^2^improvement shows in lower score,

### Factors associated to rehabilitation outcomes

The results of the bivariate and multivariate logistic regression analyses with the ADL outcomes are presented in Table 3. Having an *orthopaedic condition in the upper extremities and spine* was associated with increased functioning in ADL after rehabilitation. The relationship was slightly attenuated in the multivariate model that included gender, age, living situation, number of training sessions and length in weeks and was no longer statistically significant (*p* = 0.052). The result also points to an association between *orthopaedic conditions (lower extremities and pelvis)* (OR 2.58; 95% CI .99–6.71) and an increase in ADL after intervention, although not statistically significant at 5% level (Table 3).

**Table 3:** Factors associated with an increase in ADL outcome at 2-month follow-up

Variables	Bivariate logistic regression (***n*** = 155)		Multivariate logistic regression (***n*** = 155)	
	OR	95% CI	OR	95% CI
Gender (reference category Female = 0)				
Male	.58	.29–1.15	.71	.32–1.60
Age	.99	.96–1.03	.98	.93–1.02
Living situation (reference category Living alone = 0)				
Cohabiting	.80	.41–1.54	.96	.43–2.17
Training sessions per week by OT	1.19	.65–2.17	1.30	.62–2.72
Training sessions per week by PT	.98	.61–1.59	.87	.45–1.69
Length of home rehabilitation in weeks	.90	.79–1.02	.90	.79–1.04
Main reason for home rehabilitation (reference category Multimorbidity and/or frailty = 0)				
Neurological conditions (excl. stroke)	.32	.03–3.33	.31	.03–3.76
Stroke (both acute and post)	1.78	.63–5.07	1.85	.63–5.45
Orthopaedic conditions (upper extremities and spine)	4.41^*^	**1.07–18.09**	4.14	.95–18.01
Orthopaedic conditions (lower extremities and pelvis)	2.58	.99–6.71	2.62	.94–7.33
Mobility limitations incl. fall risk (condition not specified)	.71	.20–2.47	.73	.20–2.73
Conditions in the circulatory and respiratory systems	3.31	.91–12.06	2.83	.74–10.85

**Abbreviations:** OR, Odds Ratio; 95% CI, 95% Confidence Interval; OT, occupational therapist; PT, physiotherapist

**P-value:** *p < 0.05

Displayed in Table 4, the bivariate logistic regression analysis showed that higher frequencies of training sessions per week by an occupational therapist were associated with improvement in mobility outcomes (OR 2.09, 95% CI 1.12–3.91). The corresponding result for training sessions per week by a physiotherapist displayed a tendency in the same direction (OR 1.70, 95% CI .99–2.91). The relationship between training sessions by an occupational therapist and mobility functions did not remain statistically significant in the multivariate model. When it comes to living condition, the result points towards an association with increased mobility function for those living together with someone (OR 1.74, 95% CI .91–3.33), as compared to those living alone. However, this was not statistically significant at 5% level (Table 4).

**Table 4:** Factors associated with an increase in GMF mobility function outcome at 2-month follow-up

Variables	Bivariate logistic regression (***n*** = 150)		Multivariate logistic regression (***n*** = 150)	
	OR	95% CI	OR	95% CI
Gender (reference category Female = 0)				
Male	.98	.50–1.92	.82	.37–1.83
Age	1.00	.96–1.04	1.00	.96–1.05
Living situation (reference category Living alone = 0)				
Cohabiting	1.74	.91–3.33	1.90	.85–4.28
Training sessions per week by OT	2.09*	**1.12–3.91**	2.04	.94–4.42
Training sessions per week by PT	1.70	.99–2.91	1.35	.68–2.70
Length of home rehabilitation in weeks	1.06	.95–1.20	1.10	.96–1.26
Main reasons for home rehabilitation (reference category Multimorbidity and/or frailty = 0)				
Neurological conditions (excl. stroke)	1.27	.16–10.07	.98	.10–9.54
Stroke (both acute and post)	2.85	.97–8.34	2.73	.84–8.46
Orthopaedic conditions (upper extremities and spine)	2.11	.63–7.13	3.07	.80–11.74
Orthopaedic conditions (lower extremities and pelvis)	2.03	.80–5.16	2.51	.89–7.10
Mobility limitations incl. fall risk (condition not specified)	.79	.21–2.92	1.06	.26–4.25
Conditions in the circulatory and respiratory systems	.81	.25–2.58	1.11	.31–3.92

**Abbreviations:** OR, Odds Ratio; 95% CI, 95% Confidence Interval; OT, occupational therapist; PT, physiotherapist

**P-value:** *p < 0.05

Table 5 presents the bivariate and multivariate logistic regression analysis with GMF dependence upper limb as the outcome. A longer duration of home rehabilitation (number of weeks) was a factor related to improvement in upper limb function, and an association between *orthopaedic conditions (lower extremities and pelvis)* and *upper limb* function was found. Both relations remained statistically significant in the multivariate model.

**Table 5:** Factors associated with an increase in GMF upper limb function outcome at 2-month follow-up

Variables	Bivariate logistic regression (***n*** = 150)		Multivariate logistic regression (***n*** = 150)	
	OR	95% CI	OR	95% CI
Gender (reference category Female = 0)				
Male	.95	.36–2.55	.49	.13–1.79
Age	.99	.94–1.05	1.04	.96–1.11
Living situation (reference category Living alone = 0)				
Cohabiting	1.75	.67–4.57	1.55	.44–5.44
Training sessions per week by OT	.81	.34–1.95	0.61	.20–1.87
Training sessions per week by PT	.99	.49–2.01	1.54	.55–4.31
Length of home rehabilitation in weeks	1.26^*^	**1.04–1.54**	1.28*	**1.02–1.60**
Main reason for home rehabilitation (reference category Multimorbidity and/or frailty = 0)				
Neurological conditions (excl. stroke)	3.86	.46–32.42	7.12	.60–84.22
Stroke (both acute and post)	.70	.18–2.71	.58	.14–2.43
Orthopaedic conditions (upper extremities and spine)	.89	.20–4.01	.80	.14–4.47
Orthopaedic conditions (lower extremities and pelvis)	.10^*^	**.01–.87**	.08^*^	**.01–.73**
Mobility limitations incl. fall risk (condition not specified)	.70	.13–3.92	.57	.08–3.91
Conditions in the circulatory and respiratory systems	.23	.03–2.01	.27	.03–2.71

**Abbreviations:** OR, Odds Ratio; 95% CI, 95% Confidence Interval; OT, occupational therapist; PT, physiotherapist

**P-value:** *p < 0.05

The results of the bivariate and multivariate logistic regression analyses with self-rated health as the outcome are presented in Table 6. No statistically significant associations were found between the independent variables and self-rated health. No statistically significant interaction was found between number of training sessions and duration of intervention (number of weeks) in the logistic regression models (not displayed).

**Table 6:** Factors associated with an increase in EQ5D VAS outcome at 2-month follow-up

Variables	Bivariate logistic regression (***n*** = 97)		Multivariate logistic regression (***n*** = 97)	
	OR	95% CI	OR	95% CI
Gender (reference category Female = 0)				
Male	1.29	.52–3.17	1.00	.32–3.15
Age	.97	.92–1.03	.97	.91–1.03
Living situation (reference category Living alone = 0)				
Cohabiting	.75	.33–1.73	.56	.19–1.66
Training sessions per week by OT	.74	.36–1.53	.43	.15–1.23
Training sessions per week by PT	1.35	.69–2.65	1.92	.71–5.21
Length of home rehabilitation in weeks	.92	.78–1.08	.93	.77–1.11
Main reason for home rehabilitation (reference category Multimorbidity and/or frailty = 0)				
Neurological conditions (excl. stroke)	.69	.04–12.57	.84	.04–17.26
Stroke (both acute and post)	1.66	.43–6.38	1.65	.41–6.69
Orthopaedic conditions (upper extremities and spine)	.83	.19–3.58	.54	.11–2.70
Orthopaedic conditions (lower extremities and pelvis)	.88	.28–2.81	8	.22–2.77
Mobility limitations incl. fall risk (condition not specified)	4.15	.42–40.66	3.08	.27–35.01
Conditions in the circulatory and respiratory systems	3.81	.68–21.47	2.43	.39–15.34

**Abbreviations:** OR, Odds Ratio; 95% CI, 95% Confidence Interval; OT, occupational therapist; PT, physiotherapist

**P-value:** *p < 0.05

## Discussion

In this study we investigated changes in functioning and self-rated health in relation to a home rehabilitation program. We also investigated how socio-demographic factors, health conditions and intensity and duration of home rehabilitation interventions were associated to change in functioning and self-rated health after home rehabilitation. We found improvements in functioning and self-rated health after a home rehabilitation program and at 2-month follow-up. This is in line with several systematic reviews of home rehabilitation programs [31–35]. We found an association between higher frequencies of training sessions per week by an occupational therapist and improved mobility functions. This is in line with a study of stroke patients that found a positive correlation between the amount of training in minutes led professionals and outcomes in both ADL, motor functions and quality of life [36]. These indicate that the amount of training with professionals is a factor that needs to be considered when planning rehabilitation interventions, as it may have an impact on outcomes. Studies in Sweden have however shown that home rehabilitation interventions by occupational therapists and physiotherapists in Sweden consist of an average of five visits during a six-week period, which is a lower intensity than the home rehabilitation program in our study [14]. Our results would suggest that increasing in the intensity of professional training sessions in home rehabilitation may be necessary in clinical practice.

We also found an association between longer duration of training in weeks and improved upper limb functions. Although home rehabilitation programs are usually described as intensive interventions during a fixed duration [8, 15], there are definitions of home rehabilitation, such as in reablement, that emphasize multiple visits rather than the intervention’s time frame [16]. There are also few studies analysing the duration and frequency of training in home rehabilitation programs [33] and the time frame used in previous studies have varied from 4 to 12 weeks to 6 months [8, 15, 34, 35, 37]. Consequently, there is no consensus on the intensity and duration of home rehabilitation programs at present. To guide work in practical settings, more knowledge is needed to identify the duration and intensity where home rehabilitation programs are most effective [31, 33] as well as the possible relations to different outcomes.

In our study we found that intensive occupational therapy was a factor associated with improved mobility functions, and intensive physiotherapy also demonstrated a tendency in the same direction. Home rehabilitation teams usually vary in composition, with professionals from health- and social care [16]. Research suggests the inclusion of occupational therapists and physiotherapists [38], which is supported by our results. Interventions made by occupational therapists such as housing adaptations and provisions of mobility devices may explain why occupational therapy rather than physiotherapy was associated with mobility outcomes in our study [14]. However, more research is needed regarding outcomes related to specific professions [13, 39, 40].

Previous studies have shown that gender [20, 32], age [32] and diagnosis or condition [20] could affect the outcome of home rehabilitation programs. In our study, the participants’ gender, age or living situation were not associated with their rehabilitation outcomes. However, we found that participants with *orthopaedic conditions in upper extremities and spine* were significantly more likely to improve in ADL than the other participants, indicating some benefits for that client group in receiving home rehabilitation. An explanation for their improvement could be that these conditions often are associated with fewer residual conditions compared to e.g., stroke or multimorbidity.

While home rehabilitation programs generally should not be limited in terms of participants’ age or condition [16], some programs only include older adults (> 65) with home care services [31] and exclude persons with complex care needs (> 15 h home care per week) [5]. In Sweden where our study was conducted, home rehabilitation can be provided regardless of age or receipt of home care services [14]. Our study also included many different diagnoses and conditions in the analysis, while other studies only focus on a few of these. Further research is needed to determine whether specific client groups living in the community can benefit from intervention more than others, to guide policymaking and needs assessment. More knowledge is also needed on socio-demographic factors and health conditions, and how these affect outcomes to further improve home rehabilitation programs.

### Strength and limitations

In this study, we analysed de-identified data from a real-life setting with a sample varying in age, gender, living situations and health conditions. This contributes to the strength and relevance of our study when results are implemented in practice. Our results can also be externally generalized to similar contexts.

The study sample was rather small yielding low precision in the estimated effect measures with wide confidence intervals. Furthermore, no control group was included. Together, this prevents us from drawing definitive conclusions, even though our results point to several implications for research and practice.

A strength of our study is that the data were collected with accepted and validated instruments. However, for older people with minor functional limitations, the GMF shows ceiling effects [28] which may have affected the outcomes. Another limitation in this study is that the occupational therapists and physiotherapists who performed the interventions were also those who collected the data. The relationship between rehabilitation staff and participants could have affected the objectivity of the data measurement. This being said, the municipal improvement project included clear and structured routines for data collection and data handling to ensure the reliability of the data. The home rehabilitation interventions were not designed to be standardised but were tailored to the individual’s goals, needs and context, using a person-centred approach. Consequently, it is difficult to know if and how other factors that were not measured could have affected the results of the intervention.

## Conclusion

Overall improvements in functioning and self-rated health were found after a home rehabilitation program in a population of community-dwelling citizens. The result indicates that duration, intensity of occupational therapy and the main condition for rehabilitation may have an impact on the rehabilitation outcomes. Increased frequency of training sessions and longer duration of the program with professional rehabilitation staff, is vital to consider when implementing home rehabilitation programs. More research on home rehabilitation programs in different contexts, using a large sample and control groups is needed to further guide clinical practice and policymaking.

## Data Availability

The data that support the findings of this study were collected within a project run by the municipality of Jönköping, Sweden. Strong restrictions apply to the availability of these data, which were used under license for the current study, and so are not publicly available. Data used for this study could however be available from the authors upon reasonable request and if permission from the municipality of Jönköping is granted.
